# PGE2-EP2/EP4 signaling elicits mesoCAR T cell immunosuppression in pancreatic cancer

**DOI:** 10.3389/fimmu.2023.1209572

**Published:** 2023-06-30

**Authors:** Behnia Akbari, Tahereh Soltantoyeh, Zahra Shahosseini, Farhad Jadidi-Niaragh, Jamshid Hadjati, Christine E. Brown, Hamid Reza Mirzaei

**Affiliations:** ^1^ Department of Medical Immunology, School of Medicine, Tehran University of Medical Sciences, Tehran, Iran; ^2^ Department of Medical Biotechnology, School of Allied Medical Sciences, Iran University of Medical Sciences, Tehran, Iran; ^3^ Virology Department, Pasteur Institute of Iran, Tehran, Iran; ^4^ Department of Immunology, Faculty of Medicine, Tabriz University of Medical Sciences, Tabriz, Iran; ^5^ Department of Hematology & Hematopoietic Cell Transplantation, City of Hope Medical Center, Duarte, CA, United States; ^6^ Department of Immuno-Oncology, City of Hope Beckman Research Institute, Duarte, CA, United States; ^7^ Department of Genetics, University of Pennsylvania Perelman School of Medicine, Philadelphia, PA, United States; ^8^ Institute for Immunology and Immune Health, University of Pennsylvania Perelman School of Medicine, Philadelphia, PA, United States

**Keywords:** mesoCAR T cell, pancreatic cancer, pharmacological targeting, prostaglandin E2, EP2, EP4

## Abstract

**Introduction:**

For many years, surgery, adjuvant and combination chemotherapy have been the cornerstone of pancreatic cancer treatment. Although these approaches have improved patient survival, relapse remains a common occurrence, necessitating the exploration of novel therapeutic strategies. CAR T cell therapies are now showing tremendous success in hematological cancers. However, the clinical efficacy of CAR T cells in solid tumors remained low, notably due to presence of an immunosuppressive tumor microenvironment (TME). Prostaglandin E2, a bioactive lipid metabolite found within the TME, plays a significant role in promoting cancer progression by increasing tumor proliferation, improving angiogenesis, and impairing immune cell’s function. Despite the well-established impact of PGE2 signaling on cancer, its specific effects on CAR T cell therapy remain under investigation.

**Methods:**

To address this gap in knowledge the role of PGE2-related genes in cancer tissue and T cells of pancreatic cancer patients were evaluated *in-silico*. Through our *in vitro* study, we manufactured fully human functional mesoCAR T cells specific for pancreatic cancer and investigated the influence of PGE2-EP2/EP4 signaling on proliferation, cytotoxicity, and cytokine production of mesoCAR T cells against pancreatic cancer cells.

**Results:**

*In-silico* investigations uncovered a significant negative correlation between PGE2 expression and gene signature of memory T cells. Furthermore, *in vitro* experiments demonstrated that the activation of PGE2 signaling through EP2 and EP4 receptors suppressed the proliferation and major antitumor functions of mesoCAR T cells. Interestingly, the dual blockade of EP2 and EP4 receptors effectively reversed PGE2-mediated suppression of mesoCAR T cells, while individual receptor antagonists failed to mitigate the PGE2-induced suppression.

**Discussion:**

In summary, our findings suggest that mitigating PGE2-EP2/EP4 signaling may be a viable strategy for enhancing CAR T cell activity within the challenging TME, thereby improving the efficacy of CAR T cell therapy in clinical settings.

## Introduction

Pancreatic ductal adenocarcinoma (PDAC), the most prevalent form of pancreatic cancer, is associated with a highly unfavorable prognosis and poor overall survival rates (1, 2). Recent data indicate an increasing prevalence of PDAC, with over 60% of patients presenting with advanced metastatic disease and a median overall survival ranging from 8 to 11 months under current chemotherapeutic regimens (3, 4). Despite localized tumor presentation, the majority of patients eventually progress to metastatic disease. Thus, the development of effective systemic therapies is crucial for improving clinical outcomes in pancreatic cancer patients.

Immunotherapeutic approaches have emerged as a unique treatment option in various solid and hematologic cancers. A promising immunotherapeutic approach involves T cells engineered to express chimeric antigen receptors (CARs), which have shown remarkable clinical utility in treating hematological malignancies (5). Notably, several clinical trials utilizing CAR T cells in solid tumors, including pancreatic cancer, have reported promising therapeutic outcomes (6, 7). Among the different types of CAR T cells developed for solid tumors, mesoCAR T cells have demonstrated specific killing ability against pancreatic tumor cells in both *in vitro* and *in vivo* settings (8). These mesoCAR T cells are designed to target mesothelin (MSLN), a tumor-associated antigen with minimal or negligible expression in healthy cells, making it an ideal target for CAR T cell therapy in pancreatic cancer (8, 9). However, despite the development of mesoCAR T cells, their clinical success in treating pancreatic cancer has been modest, largely due to the highly immunosuppressive tumor microenvironment (TME) (10). The TME contains various metabolic immunosuppressive molecules produced by stromal cells, tumor cells, and infiltrating immune cells, which contribute to immune suppression (11, 12).

Prostaglandins (PGs), bioactive lipid metabolites generated from arachidonic acid by key enzymes such as cyclooxygenases (COXs) and PGE synthases, play a significant role in suppressing the antitumor immune response (11, 13). In fact, PG synthesis inhibitors, such as nonsteroidal anti-inflammatory drugs (NSAIDs), have demonstrated prophylactic and therapeutic advantages in cancer patients (14). Among the PGs, prostaglandin E2 (PGE2) is the most abundant in several tumors, particularly in pancreatic cancer (15–17). PGE2 exerts its functions through four G protein-coupled receptors: EP1, EP2, EP3, and EP4 (18). PGE2 has been shown to promote tumor cell proliferation, invasiveness, and tumor-associated angiogenesis, while also reprogramming myeloid cells into tumor-associated macrophages (TAMs) with an M2 phenotype. Furthermore, PGE2 suppresses the production of interferon-gamma (IFN-γ) by Natural Killer cells (NK cells) and T cells (19–21). Among the four cognate receptors of PGE2, EP2 and EP4 receptors, which increase intracellular cyclic AMP (cAMP) and protein kinase A (PKA) phosphorylation upon PGE2 ligation, have been implicated in cancer development and the suppression of antitumor immune responses (22, 23). While previous studies have provided insights into the potential actions of PGE2 in the TME and its effects on T cells, they have neither revealed the association between PG-related genes expression and PDAC development and patients’ survival nor addressed the role of PGE2 signaling on antitumor function of mesoCAR T cells in context of pancreatic cancer.

To address these questions, we investigated the effects of PGE2 on patient survival and identified correlations between PGE2-related gene expression and different T cell phenotypes. Additionally, to simulate PGE2-mediated immunosuppression within the TME, we cultured T cells and mesoCAR T cells in presence of PGE2 and evaluated the immunosuppressive effects of PGE2 on these cells. Lastly, we assessed the impact of pharmacologically targeting PGE2-mediated immunosuppression using specific PGE2 receptor antagonists on the function of mesoCAR T cells *in vitro*.

## Material and methods

### Bioinformatics analyses

Differentially expressed genes (DEGs) were extracted from the Cancer Genome Atlas (TCGA) dataset using the GEPIA2 database (http://gepia2.cancer-pku.cn) (24). The DEGs in pancreatic ductal adenocarcinoma were obtained by applying the “Differential Genes” module of GEPIA2, with the dataset set to PAAD and the differential method set to ANOVA. VolcaNoseR was used to visualize the extracted DEGs (25). GEPIA2 was also utilized to determine Overall Survival and Disease-Free Survival of PAAD patients. The “Survival Analysis” module was employed with Group Cutoff set to the median. Hazard ratios (HRs) with 95% confidence intervals (CIs) and log-rank P-values were calculated. The “Correlation Analysis” module of GEPIA2 was employed to identify the correlation between the gene signature of different phenotypes of T cells and PGE-related genes, using tumor and normal TCGA datasets for PDAC patients. The HPA (Human Protein Atlas “proteinatlas.org”) database was used to obtain the single-cell expression of PGE receptors (EP2/EP4) in different cells under physiological conditions (26).

### Cell lines

HEK293T, Jurkat, AsPC-1, and PANC-1 cells were obtained from the Iranian Biological Resource Center (IBRC). HEK293T and PANC-1 cells were cultured in D10 media, composed of DMEM (Gibco, Life Technologies), 10% fetal bovine serum (FBS), and 1% penicillin/streptomycin (Gibco, Life Technologies). AsPC-1 and Jurkat cells were cultured in R10 media, consisting of RPMI-1640 (Gibco, Life Technologies) supplemented with 10% FBS, 25 mM HEPES (Sigma Aldrich), 2 mM glutamine (Gibco), and 1% penicillin/streptomycin. Prior to experiments, mesothelin expression was authenticated by flow cytometry on the relevant cell lines. All cell lines were regularly tested for mycoplasma contamination.

### Lentiviral vector production

Lentiviral vectors were produced as previously described (27). Briefly, HEK293T cells were transfected with lentiviral CAR and packaging plasmids using the calcium phosphate method. Lentiviral supernatants were collected at 48- and 72-hours post-transfection and concentrated through high-speed centrifugation. The resulting concentrated lentivirus batches were then resuspended in cold RPMI-1640 media and stored at -80°C. Lentiviral vectors were titrated using Jurkat cells.

### T cell isolation and CAR T cell manufacturing

Healthy donor white blood cells were obtained from the Iranian Blood Transfusion Organization (IBTO). Peripheral blood mononuclear cells (PBMCs) were isolated using standard methods with Histopaque^®^-1077 (Sigma Aldrich). T cells were negatively selected using immunomagnetic beads (Pan T Cell Isolation Kit, Miltenyi Biotec) and stored at -80°C. For mesoCAR T cell production, 1 × 10^6^ T cells were seeded in each well of 12-well Costar tissue culture plates and activated using Dynabeads™ Human T-Expander CD3/CD28 (Gibco, Life Technologies, 11161D) at a 1:1 ratio in TM10 media, which consisted of TexMACS™ Medium (Miltenyi Biotec) supplemented with 10% AB serum and 100 IU/mL premium-grade rhIL-2 (Miltenyi Biotec). Twenty-four hours post-activation, lentiviral vectors supplemented with 8 μg/mL Polybrene (Santacruz) were added to the early activated T cells at a multiplicity of infection (MOI) of five. Centrifugation at 850g for 1 hour at 32°C was performed to enhance transduction efficacy. Two hours after centrifugation, 2 mL/well of TM10 media were added to the transduced T cells. At day 4 post-transduction, Dynabeads™ were removed from the transduced T cells using a DynaMag™ magnet, and GFP expression, as a representative of mesoCAR expression, was assessed using flow cytometry.

### PGE2, PF-04418948, and E7046 dose-response

To determine the most effective concentration of PGE2 (MedChemExpress), PF-04418948 (MedChemExpress), and E7046 (Cayman Chemical), 1 × 10^5^ CFSE-labeled T cells were seeded in 96-well Costar tissue culture plates and cultured with various doses of PGE2, PF-04418948, and E7046. The cells were activated with Dynabeads™ Human T-Expander CD3/CD28 (Gibco, Life Technologies, 11161D) at a 1:1 ratio in TM10 media. After three days, T cells were harvested, and their proliferation was determined using flow cytometry.

### *In vitro* cytotoxicity assay

mesoCAR T cells and untransduced T cells were co-incubated at 1:1, 5:1, 10:1, and 30:1 ratios with 1 × 10^4^ CFSE stained target cells for 4 hours in TM10 media in 96‐well U‐bottomed plates, with a final volume of 200 μl/well. To distinguish between effector and target cells, the cell suspension was harvested and stained with anti-human CD3 conjugated with APC (Clone: UCHT1, BioLegend). To stain for dead cells, 7-AAD (Miltenyi Biotec) was added 30 minutes before flow cytometry. Flow cytometry analysis was performed using CFSE, CD3 and 7-AAD staining to distinguish T cells from dead tumor cells. The frequency of lysed target cells (CFSE+/CD3-/7-AAD+ cells) was calculated by subtracting the percentage of spontaneous lysis of target cells from the percentage of target cells in coculture with mesoCAR T cells. Specific lysis was reported by normalizing target cell lysis based on the expression of mesothelin on target cells.

### *In vitro* proliferation and cytokine production assays

Target cells were treated with 50 μg/ml of mitomycin C (Sigma Aldrich) for 30 minutes at 37°C and extensively washed (28). To track cell proliferation using CFSE dye (Life Technologies), mesoCAR T cells and untransduced T cells (1.2 × 10^7^/ml) were stained with 5 μM CFSE at room temperature for 8 minutes. The reaction was terminated by adding an equal amount of FBS. After washing three times with complete RPMI 1640 medium, CFSE-labeled cells (0.2 × 10^6^/well) were cocultured at a 1:1 ratio with either target cells or cultured in media (without target cells) in the absence of exogenous IL‐2 in 48‐well plates, with a final volume of 800 μl/well. The supernatant was harvested 24 hours after plating and stored at −20°C until subsequent cytokine analysis by enzyme‐linked immunosorbent assay (ELISA) to quantify IFN‐γ and IL‐2. After 72 hours, cells were stained with anti‐CD3‐APC (Clone: UCHT1, BioLegend), and CFSE dilution of CD3+ cells, as a measure of proliferation, was determined by flow cytometry, as previously described (29).

### Flow cytometric analysis

To check the purity of isolated T cells using magnetic beads, isolated T cells were stained with APC-conjugated anti‐human CD3 (Clone: UCHT1, BioLegend). Mesothelin expression was detected using PE-conjugated anti-human mesothelin (Clone: #420411, R&D Systems). All samples were acquired with a BD FACSCalibur (BD Biosciences) and analyzed using FlowJo software (v10.6). All assays were performed in duplicate and repeated two to three times.

### Statistical analysis

Normality tests, one-way and two-way analysis of variance (ANOVA), were used to identify possible differences among different treatment groups using GraphPad Prism software (v9). p-values less than 0.05 were considered statistically significant.

## Results

### Prostaglandin E2 and its receptors may modulate T cell responses in pancreatic cancer patients

**Figure 1:**
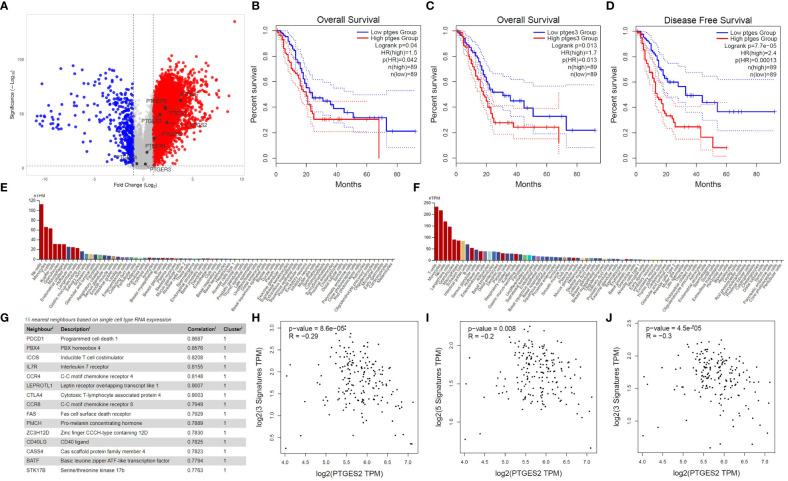
Bioinformatics investigation of PGE2 genes in pancreatic cancer. **(A)** Volcano plot illustrating the differential expression of genes in pancreatic cancer patients compared to healthy samples, with a focus on PGE2-related genes. **(B, C)** Kaplan-Meier survival curves showing the overall survival time of patients with high expression of PTGES and PTGES3 compared to patients with low expression. **(D)** Kaplan-Meier survival curve demonstrating the disease-free survival time in patients with high expression of PTGES compared to patients with low expression. Dotted lines represent the 95% confidence interval in the Kaplan-Meier plots. **(E, F)** Immunohistochemistry data from the Human Protein Atlas (HPA) showing high expression of EP2 and EP4 receptors on T cells. **(G)** List of genes showing a significant correlation with EP4 receptor expression in T cells. **(H–J)** Scatter plots depicting the negative correlation between PTGES2 expression and gene signatures of central memory **(H)**, effector memory **(I)**, and effector T cells **(J)** in pancreatic cancer patients.

To investigate the significance of PG-related genes in PDAC, we utilized Gepia2 and VolcaNoseR to extract and visualize differentially expressed genes (DEGs). Our data indicate that prostaglandin E synthase and prostaglandin-endoperoxide synthase genes, including PTGES, PTGES2, PTGES3, PTGS1, and PTGS2, are highly enriched in PDAC patients (**Figure 1A**). However, among the prostaglandin receptors, only the PTGER2 (EP2) gene showed upregulation, while PTGER1, PTGER3, and PTGER4 exhibited no significant change in expression in PDAC patients. Next, to determine the prognostic value of PG-related genes, we conducted Kaplan-Meier survival analyses using Gepia2. Our findings suggest that elevated expression of prostaglandin synthesis enzymes is an unfavorable prognostic marker for PDAC patients. Specifically, high expression of PTGES and PTGES3 significantly decreases overall survival of patients (**Figures 1B, C**). Moreover, high PTGES expression significantly decreases disease-free survival of patients (**Figure 1D**). Although not statistically significant, patients with higher expression of PTGS1 and PTGS2 genes, as well as genes corresponding to prostaglandin receptors, showed decreased overall survival and disease-free survival (**Figures S1A–S1L**).

To further understand the role of prostaglandin E signaling in immune cells, we utilized single-cell RNA-seq data from the Human Protein Atlas. According to these data, EP1 and EP3 receptors have no to low expression in T cells (Data not shown). However, EP2 and EP4 are highly expressed in immune cells, especially T cells (**Figures 1E, F**), with EP4 being part of a cluster related to T cell immune response and showing the highest correlation (0.8687) with the PD1 gene (**Figure 1G**). Based on single-cell RNA-seq data, ICOS, IL7R, CCR7, CTLA4, CCR8, and FAS are also immunologically important genes in the neighborhood of the EP4 receptor (**Figure 1G**). Previous studies have indicated that PGE2 ligation with EP2 and EP4 receptors can promote immunosuppression in T cells by inhibiting IL-2 production, reducing CD25 expression, and, most importantly, impairing IFN-γ secretion and T cell effector function (11, 23). Therefore, we conducted several correlation analyses to elucidate the role of prostaglandin E2 receptor signaling in promoting T cell immunosuppression. Our results demonstrate that PGE2 expression is negatively correlated with the gene signature of central memory (**Figure 1H**) and effector memory (**Figure 1I**) T cells. This negative correlation was also observed in the gene signature related to effector T cells (**Figure 1J**). Interestingly, a significant positive correlation was observed between the gene signatures of central memory, effector memory, and exhausted T cells with EP2 and EP4 receptors (**Figure S2**). These data collectively indicate that PGE2 and its immunoinhibitory receptors (EP2 and EP4) can possibly modulate T cell responses in PDAC patients.

### PGE2 suppresses T cell proliferation through EP2/EP4 signaling

**Figure 2:**
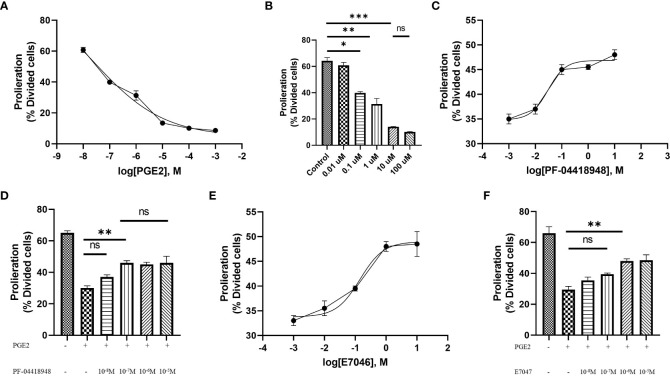
Antigen-independent proliferation of T cells in culture with PGE2 and EP2/4 antagonists. **(A)** Dose-response analysis of T cell proliferation mediated by CD3/28 stimulation in the presence of various concentrations of PGE2. **(B)** Significant inhibition of T cell proliferation by PGE2 at concentrations of 0.1, 1, and 10 µM. **(C)** Dose-response analysis of T cell proliferation in the presence of 10 µM PGE2 and different concentrations of PF-04418948, an EP2/4 antagonist. **(D)** Significant reversal of PGE2-mediated suppression of T cell proliferation by PF-04418948 at a concentration of 0.1 µM. **(E)** Dose-response analysis of antigen-independent proliferation of T cells in the presence of 10 µM PGE2 and various concentrations of E7046, an EP4 antagonist. **(F)** Significant enhancement of T cell proliferation by 1 µM E7046 in the presence of 10 µM PGE2. Statistical analysis was performed using ordinary one-way ANOVA and Tukey multiple comparison test. *P < 0.05; **P < 0.01; ***P < 0.001. Data are presented as mean ± SD.

T cell expansion and proliferation are key determinants of successful cellular therapy in clinical settings (30). Previous reports have demonstrated that low concentrations of PGE2 are essential for T cell activation and differentiation (31), whereas higher concentrations of PGE2 (>1 µM), commonly found at the tumor site, induce subversion of CD8 differentiation, suppression of T cell proliferation, and inhibition of CD4 T cell helper functions (23). Therefore, to understand the function of PGE2 on T cell proliferation through EP2 and EP4 receptor signaling and determine suitable doses for our study, we exposed T cells activated with anti-CD3/CD28 coated beads to different concentrations of PGE2 and orally available EP2 and EP4 antagonists, PF-04418948 and E7046, respectively. Our findings suggest that PGE2 can decrease antigen-nonspecific T cell proliferation in a dose-dependent manner, with 10 µM of PGE2 demonstrating maximal efficacy in inhibiting T cell proliferation (**Figures 2A, B**).

Next, to determine if pharmacological blockade of EP2 and EP4 receptors can diminish the inhibitory effects of PGE2, we used different concentrations (ranging from 0.01 µM to 10 µM) of PF-04418948 or E7046 in cultures of T cells treated with 10 µM of PGE2 and activated by anti-CD3/CD28 coated beads. Interestingly, pharmacological blockade of these receptors enhances T cell proliferation in a dose-dependent manner. Based on dose-response analyses, a 0.1 µM dose of PF-04418948 (**Figures 2C, D**) and a 1 µM dose of E7047 (**Figures 2E, F**) show maximal efficacy in enhancing T cell proliferation. However, single pharmacological blockade of these receptors fails to fully eliminate the inhibitory function of PGE2 on antigen-nonspecific T cell proliferation.

### Manufacturing and functional characterization of fully human MesoCAR T cells against pancreatic cancer cell lines

**Figure 3:**
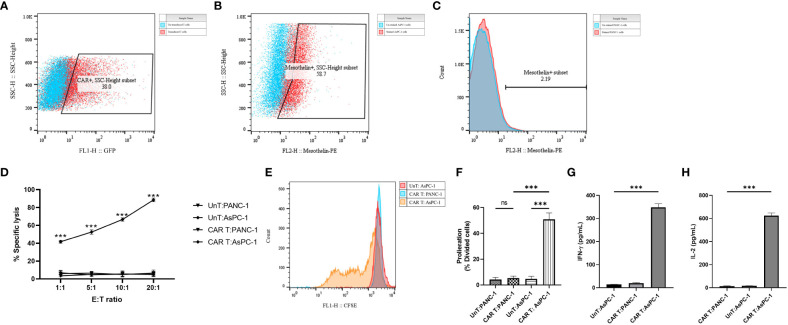
Antigen specificity of mesoCAR T cells against pancreatic cancer cells. **(A)** Flow cytometry dot plot demonstrating the expression of the chimeric antigen receptor (CAR) on mesoCAR T cells after manufacturing. **(B, C)** Representative dot and histogram plots showing the mesothelin expression on AsPC-1 and PANC-1 cells, respectively. **(D)** Specific lysis of target cells by mesoCAR T cells at different effector-to-target ratios. **(E, F)** Proliferation of mesoCAR T cells in response to target cells. **(G, H)** Production of IFN-γ and IL-2 by mesoCAR T cells in coculture with target cells. Statistical analysis was performed using ordinary one-way ANOVA **(E–H)** and two-way ANOVA **(D)** followed by Tukey multiple comparison test. ***P < 0.001. Data are presented as mean ± SD.

Primary human CD3+ T cells were efficiently infected with replication-defective lentiviral particles encoding the second-generation mesoCAR transgene at an MOI of 5, with reproducible transduction efficacy of approximately 30% (**Figure 3A**). To further characterize and measure the *in vitro* antitumor capacity of the produced mesoCAR T cells, we utilized PANC-1 and AsPC-1 cells as mesothelin-negative and positive pancreatic cancer cells in our experiments (**Figures 3B, C**).

The cytolytic abilities of T cells expressing the mesoCAR transgene were evaluated using a 4-hour CD3/7AAD-based cytotoxicity assay. Genetically modified mesoCAR T cells specifically lysed mesothelin+ AsPC-1 cells. We observed antigen-specific lysis of AsPC-1 cells even at an E:T ratio as low as 1:1 (**Figure 3D**). Lysis of PANC-1 cells by mesoCAR T cells and lysis of AsPC-1 cells by untransduced T cells were not detected, demonstrating the antigen specificity of the cytolysis and the lack of natural activity of the generated mesoCAR T cells (**Figure 3D**). T cell proliferation and cytokine production are two other key components in the generation of a robust and sustained antitumor immune response. To assess whether the designed mesoCAR T cells can proliferate and produce cytokines against pancreatic cancer cells, we investigated the proliferation capacity of mesoCAR T cells and their production of IL-2 and IFN-γ compared to untransduced T cells upon antigen-specific stimulation *in vitro*. Following CAR T cell restimulation with AsPC-1 and PANC-1 cells, T cells expressing the mesoCAR exhibited a significantly high mesothelin-specific proliferation rate comparable to untransduced T cells stimulated *via* the endogenous TCR (**Figures 3E, F**). Cytokine measurements using ELISA following mesoCAR activation with AsPC-1 cells revealed that CAR T cells produce large quantities of IFN-γ and IL-2, comparable to untransduced T cells (**Figures 3G,H**). No IL-2 and IFN-γ secretion was detected in cultures of T cells or tumor cells alone or irrelevant target cells (PANC-1). This shows that T cell activation through the mesoCAR could lead to the induction of mesothelin-specific IL-2 and IFN-γ production. The cytokine production pattern aligns with the Th1-like phenotype of T cells generated by anti-CD3 and CD28-coated beads and, thereby, supports an effective antitumor cellular immune response (32).

### PGE2 signaling through EP2/EP4 receptors diminishes MesoCAR T cell antitumor function

**Figure 4:**
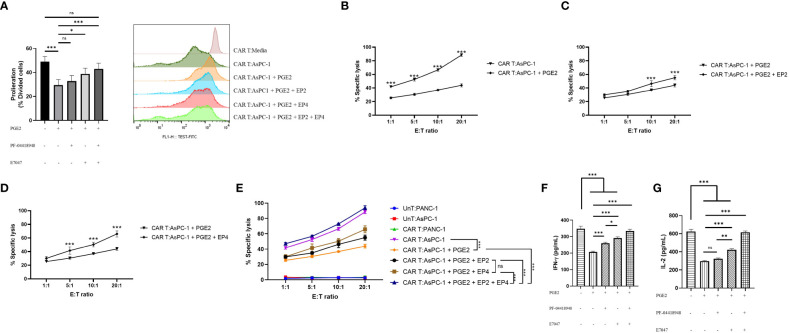
The effects of PGE2-EP2/EP4 signaling on the antitumor function of mesoCAR T cells. **(A)** Antigen-specific proliferative capacity of mesoCAR T cells over a three-day period in the presence of PGE2 alone or in combination with EP2 and/or EP4 antagonists. **(B)** Specific lysis of AsPC-1 cells by mesoCAR T cells in the presence of 10 µM PGE2. **(C, D)** Cytotoxic function of mesoCAR T cells against AsPC-1 cells after blockade of EP2 **(C)** and EP4 **(D)** receptors. **(E)** Overlaid plot demonstrating mesoCAR T cell cytotoxicity against AsPC-1 cells in the presence of PGE2 alone or in combination with EP2 and/or EP4 antagonists. **(F, G)** Production of IFN-γ and IL-2 by mesoCAR T cells in coculture with AsPC-1 cells in the presence of PGE2 alone or in combination with EP2 and/or EP4 antagonists. Statistical analysis was performed using ordinary one-way ANOVA **(A, F, G)**, two-way ANOVA **(B–E)**, and Tukey multiple comparison test. *P < 0.05; **P < 0.01; ***P < 0.001. Data are presented as mean ± SD.

To investigate the role of PGE2 in antigen-specific immunosuppression, we aimed to measure the proliferation capacity, cytotoxic function, and cytokine production of mesoCAR T cells in coculture with AsPC-1 cells using different concentrations of PGE2, PF-04418948, and E7046. Our findings suggest that PGE2 at a dose of 10 µM significantly decreases the proliferation of mesoCAR T cells. Interestingly, the addition of EP2 has no significant impact on mesoCAR T cell proliferation. Although EP4 blockade alone significantly enhances mesoCAR T cell proliferation, it fails to fully restore mesoCAR T cell proliferation (**Figure 4A**). In contrast, the double pharmacological blockade of these receptors successfully removes the inhibitory effects of PGE2 on mesoCAR T cell proliferation (**Figure 4A**).

Furthermore, PGE2 was found to inhibit the cytotoxic function of mesoCAR T cells against pancreatic cancer cells, even at low ratios such as 1:1 (**Figure 4B**). The blockade of the EP2 receptor in the presence of PGE2 was shown to slightly enhance mesoCAR T cell cytotoxicity at 10:1 and 20:1 ratios (**Figure 4C**). Interestingly, EP4 blockade was shown to improve mesoCAR T cell cytotoxicity even at low ratios (5:1), indicating that EP4 signaling is more important for mesoCAR T cell cytotoxic function against pancreatic cancer cells (**Figure 4D**). Additionally, the double blockade of EP2 and EP4 receptors was shown to completely restore the cytotoxic function of mesoCAR T cells in the presence of PGE2 (**Figure 4E**).

Lastly, it was observed that PGE2 is able to suppress both IFN-γ and IL-2 production from mesoCAR T cells (**Figures 4F, G**). Although EP2 blockade alone fails to enhance IL-2 production from mesoCAR T cells in the presence of PGE2, EP4 blockade and the double blockade of these receptors improve the production of IL-2 against tumor cells in the presence of PGE2 (**Figure 4F**). In terms of IFN-γ, EP4 targeting showed the greatest impact on IFN-γ production (**Figure 4G**). Overall, simultaneous pharmacological blockade of EP2 and EP4 receptors boosted IFN-γ and IL-2 production from mesoCAR T cells in the presence of PGE2.

## Discussion

The hostile and complex TME surrounding PDAC tumors poses a significant challenge to the effectiveness of adoptive cellular therapy. This TME is characterized by a dense desmoplastic stroma and the presence of immunosuppressive metabolites, along with extensive infiltration of immunosuppressive cells such as tumor-associated macrophages (TAMs), myeloid-derived suppressor cells (MDSCs), and regulatory T cells (33). In this study, our aim was to investigate the role of a bioactive metabolite, prostaglandin E2 (PGE2), in the context of PDAC.

Our *in-silico* analyses revealed a significant upregulation of PG-related genes in PDAC tumors, and we observed a negative correlation between the expression of Prostaglandin E Synthase (PTGES) and the survival rate of PDAC patients. Additionally, we identified a strong negative correlation between PTGE2 expression and effector and memory T cells in PDAC. We also found a strong positive correlation between the expression of the EP4 receptor and PD-1 in T cells, suggesting a potential combination therapy involving PD-1 blockade and inhibition of PGE2 signaling. Consistent with our findings, previous studies have demonstrated that high expression of PGE2 and activation of EP2/EP4 signaling not only increase PD-1 expression in T cells (34) but also promote PD-L1 expression in TAMs and MDSCs, which are highly abundant suppressive cells in PDAC (35). Further investigations revealed that PGE2 promotes the expression and secretion of ARG1 and iNOS in MDSCs, while pharmacological blockade of EP4 inhibits the secretion of these proteins, thereby inhibiting the function of MDSCs (36).. Recent studies have shown that combining EP4 blockade with anti-PD-1 immunotherapy synergistically enhances the antitumor response of tumor-specific cytotoxic T lymphocytes (CTLs) (35, 37).

Subsequently, through our sets of *in vitro* studies on mesoCAR T cells we demonstrate that PGE2 signaling through EP2 and EP4 receptors is responsible for limited antitumor function of these cells in PGE2-rich tumors. From a mechanistical point of view, EP2/EP4 downstream signaling in T cells were previously shown to initiate and activate PKA and PI3K signaling pathways, leading to cAMP accumulation and dysregulation of the AKT/mTOR pathway within T cells, respectively (11, 23). It has been previously shown that accumulation of cAMP in T cells impairs their normal function, resulting in T cell dysfunction in chronic infections and tumors (38). Moreover, reducing cAMP accumulation through targeting upstream molecules, such as the A2a receptor, has shown promising results in CAR T cells by empowering CAR T cells antitumor properties (39–41). Additionally, blocking the PKA pathway, for example through AKT inhibition, in CAR T cells has led to improved CAR T cell persistence, proliferation, and effector function both *in vitro* and *in vivo* (42–45). These results altogether suggests that mesoCAR T cell therapy in combination with EP2/EP4 antagonists can achieve improved preclinical and clinical responses in PDAC.

The presence of tumor-infiltrating T cells and CAR T cells is indicative of tumor immunosurveillance and holds significant therapeutic and prognostic relevance (46). However, the dense desmoplastic stroma, which constitutes nearly 50% of the total tumor mass in PDAC, acts as a barrier to the infiltration of antitumor immune cells such as CAR T cells (47). Notably, previous studies have demonstrated that pharmacological blockade of EP4 improves the infiltration of tumor-specific T cells into the tumor site and promotes tumor rejection in a colorectal cancer syngeneic mouse model (35). Clinical data from patients with advanced tumors showing high MDSC infiltration who received daily doses of the EP4 antagonist E7046 as monotherapy demonstrated stable disease for more than 18 weeks, increased serum levels of CXCL10 (a T cell recruiting chemokine), and improved infiltration of CD3+ cells into the tumor site (48). Single-cell data from prostate cancer patients revealed EP4 as a universal marker of T cell exhaustion, and targeting the EP4 receptor was able to restore T cell infiltration into the TME and promote the proliferation of tumor-specific T cells (36). Most recently, a dual EP2/EP4 antagonist called TPST-1495 is currently being evaluated as a single agent and in combination with pembrolizumab in a clinical trial (NCT04344795). According to preliminary and unpublished data, TPST-1495 has shown the ability to block PGE2-mediated suppression of T cells, significantly enhance IFN-γ production in response to cognate peptide antigen, reduce tumor outgrowth in mouse models of solid tumors, and increase the infiltration of cytotoxic NK cells and tumor-specific and non-specific T cells at the tumor site (49). Collectively, pharmacological blockade of these two receptors holds great promise in combination with CAR T cell therapy, particularly in immunologically cold tumors such as pancreatic cancer.

Limitations of our study include the focus on PGE2 and its interaction with specific receptors (EP2 and EP4) without considering other immunosuppressive factors and signaling pathways within the tumor microenvironment. Furthermore, through our study we primarily relied on *in vitro* experiments using cell lines and isolated T cells, which may not fully capture the complexity and dynamics of the immune system in the human body. *In vivo* validation of the findings is lacking, and the clinical relevance of the observed effects and therapeutic implications requires further investigation through preclinical and clinical studies. Finally, future studies should extensively explore potential confounding factors such as other soluble mediators, genetic variations, or the heterogeneity of T cell populations, which could influence the observed effects in our study.

## Conclusions

In conclusion, our study elucidates the roles of PGE2 and its receptors EP2 and EP4 in the immunosuppressive TME of PDAC. The findings highlight the potential of targeting PGE2 signaling pathways as a strategy to enhance the antitumor function of CAR T cells and improve therapeutic responses in PDAC. Further research is warranted to validate these findings in *in vivo* models and clinical settings, and to explore the combination of EP2/EP4 antagonists with immunotherapies such as PD-1 blockade for enhanced efficacy.

## Data availability statement

The original contributions presented in the study are included in the article/**Supplementary Material**. Further inquiries can be directed to the corresponding authors.

## Ethics statement

The studies involving human participants were reviewed and approved by the Research Ethics Committees of the School of Medicine, Tehran University of Medical Sciences [IR.TUMS.MEDICINE.REC.1399.876]. The patients/participants provided their written informed consent to participate in this study.

## Author contributions

Conception and design of studies: BA, CB, and HM. Acquisition, analysis and interpretation: BA, TS, ZS, and FJ-N. Drafting article: BA. Critical review and discussion: BA, CB, JH, and HM. All authors contributed to the article and approved the submitted version.

## Supplementary material

Supplementary Figure 1 Survival plots of PDAC patients. **(A-F).** Kaplan-Meier survival curves showing the overall survival time of patients with high expression of PTGS1 **(A)**, PTGS2 **(B)**, PTGER1 **(C)**, PTGER2 **(D)**, PTGER3 **(E)**, and PTGER4 **(F)** compared to patients with low expression. **(G-L).** Kaplan-Meier survival curve demonstrating the disease-free survival time in patients with high expression of PTGS1 **(G)**, PTGS2 **(H)**, PTGER1 **(I)**, PTGER2 **(J)**, PTGER3 **(K)**, and PTGER4 **(L)** compared to patients with low expression.

Supplementary Figure 2 Correlation between PGTER2 and PTGER4 gene expression with immune-phenotype of T cells. **(A-C).** Scatter plots depicting the correlation between PTGER2 expression and gene signatures of central memory **(A)**, effector memory **(B)**, and exhausted T cells **(C)** in pancreatic cancer patients. **(D-F).** Scatter plots depicting the correlation between PTGER4 expression and gene signatures of central memory **(D)**, effector memory **(E)**, and exhausted T cells **(F)** in pancreatic cancer patients.
