# Supplementary figures and images for: PGE2-EP2/EP4 signaling elicits mesoCAR T cell immunosuppression in pancreatic cancer
Source: Front Immunol. 2023 Jun 30;14:1209572. doi: 10.3389/fimmu.2023.1209572 (PMC10348647; doi:10.3389/fimmu.2023.1209572)

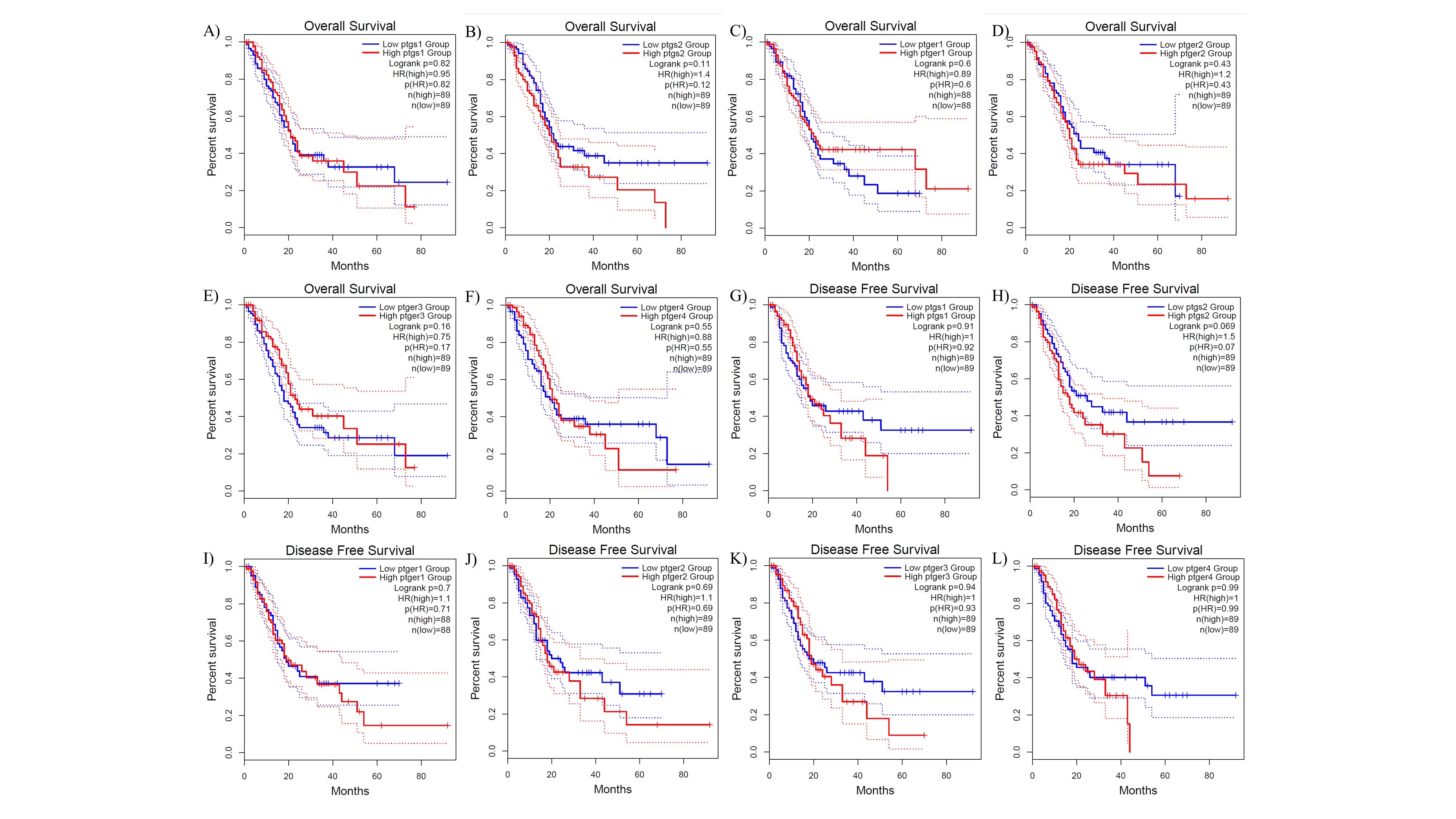

Supplement: Supplementary Figure 1 — Survival plots of PDAC patients. (A-F). Kaplan-Meier survival curves showing the overall survival time of patients with high expression of PTGS1 (A), PTGS2 (B), PTGER1 (C), PTGER2 (D), PTGER3 (E), and PTGER4 (F) compared to patients with low expression. (G-L). Kaplan-Meier survival curve demonstrating the disease-free survival time in patients with high expression of PTGS1 (G), PTGS2 (H), PTGER1 (I), PTGER2 (J), PTGER3 (K), and PTGER4 (L) compared to patients with low expression. [file Image_1.jpeg]

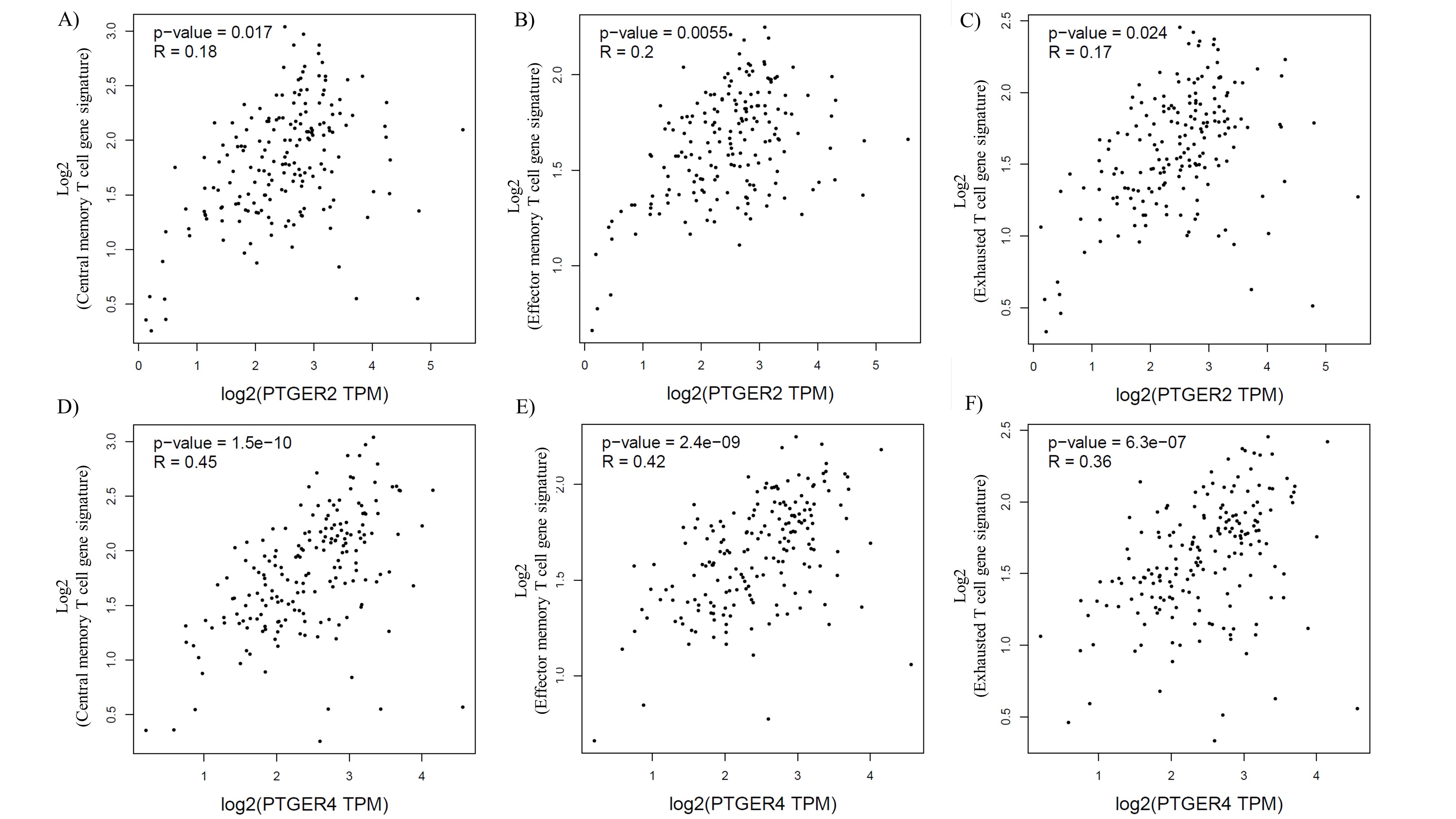

Supplement: Supplementary Figure 2 — Correlation between PGTER2 and PTGER4 gene expression with immune-phenotype of T cells. (A-C). Scatter plots depicting the correlation between PTGER2 expression and gene signatures of central memory (A), effector memory (B), and exhausted T cells (C) in pancreatic cancer patients. (D-F). Scatter plots depicting the correlation between PTGER4 expression and gene signatures of central memory (D), effector memory (E), and exhausted T cells (F) in pancreatic cancer patients. [file Image_2.jpeg]
